# PE859, a Novel Tau Aggregation Inhibitor, Reduces Aggregated Tau and Prevents Onset and Progression of Neural Dysfunction *In Vivo*

**DOI:** 10.1371/journal.pone.0117511

**Published:** 2015-02-06

**Authors:** Michiaki Okuda, Ichiro Hijikuro, Yuki Fujita, Xiaofeng Wu, Shinichi Nakayama, Yoko Sakata, Yuji Noguchi, Makoto Ogo, Shigeru Akasofu, Yoshimasa Ito, Yoshiyuki Soeda, Nobuhiko Tsuchiya, Naoki Tanaka, Takashi Takahashi, Hachiro Sugimoto

**Affiliations:** 1 Pharma Eight Co., Ltd., Kyoto, Japan; 2 Graduate School of Brain Science, Doshisha University, Kyoto, Japan; 3 ChemGenesis Inc., Tokyo, Japan; 4 Key Lab of Reproductive Biology, Institute of Zoology, Chinese Academy of Sciences, Beijing, China; 5 Tsukuba Research Laboratories, Eisai Co., Ltd., Tsukuba, Japan; 6 Department of Aging Neurobiology, Center for Development of Advanced Medicine for Dementia, National Center for Geriatrics and Gerontology, Obu, Japan; 7 Department of Biomolecular Engineering, Kyoto Institute of Technology, Kyoto, Japan; 8 Natural Product Chemistry & Pharmaceutical Research Center, Yokohama College of Pharmacy, Yokohama, Japan; Inserm U837, FRANCE

## Abstract

In tauopathies, a neural microtubule-associated protein tau (MAPT) is abnormally aggregated and forms neurofibrillary tangle. Therefore, inhibition of the tau aggregation is one of the key approaches for the treatment of these diseases. Here, we have identified a novel tau aggregation inhibitor, PE859. An oral administration of PE859 resulted in the significant reduction of sarkosyl-insoluble aggregated tau along with the prevention of onset and progression of the motor dysfunction in JNPL3 P301L-mutated human tau transgenic mice. These results suggest that PE859 is useful for the treatment of tauopathies.

## Introduction

Neurofibrillary tangle (NFT), consisting of aggregated tau proteins, is found in some neurodegenerative diseases such as Frontotemporal Dementia and Parkinsonism linked to chromosome 17 (FTDP-17), corticobasal degeneration, Pick’s disease and progressive supranuclear palsy [1, 2]. They are collectively called tauopathies. NFT is also known as one of the major pathological hallmarks of Alzheimer’s disease (AD) in concurrence with senile plaque.

Tau is one of the microtubule-associated proteins and normally binds to microtubule and plays a fundamental role in stabilization of microtubules [3, 4]. The microtubule (MT)-binding capability of tau is regulated by its phosphorylation [5]. In tauopathies and AD, hyperphosphorylated tau dissociates from microtubules, changes in conformation and self-aggregates into paired helical filament (PHF), further forming NFT [6–8]. It is thought that the aggregating process of tau correlates with neuronal dysfunction, and in fact, the severity of AD is positively related to the number of NFT [9, 10].

On the basis of these findings, several therapeutic approaches for treating neurodegenerative tauopathies have been proposed, such as, kinase inhibitors [11], microtubule stabilizer [12], tau aggregation inhibitor [13], immunotherapy [14] and chaperone-based drugs targeting disease-specific tau species [15].

In this connection, we have focused on the inhibition of tau aggregation and performed a screening for tau aggregation inhibitor in our own compound library, and identified a compound PE859 (3-[(1E)-2-(1H-indol-6-yl)ethenyl]-5-[(1E)-2-[2-methoxy-4-(2-pyridylmethoxy)phenyl]ethenyl]-1H-pyrazole). Here we show that PE859 inhibits tau aggregation *in vitro* and that an oral administration of PE859 reduces aggregated tau in the tissue of the central nervous system and delays the onset and progression of motor dysfunction in JNPL3 human P301L tau transgenic mice.

## Materials and Methods

### 2.1. Test compound

**Fig 1:**
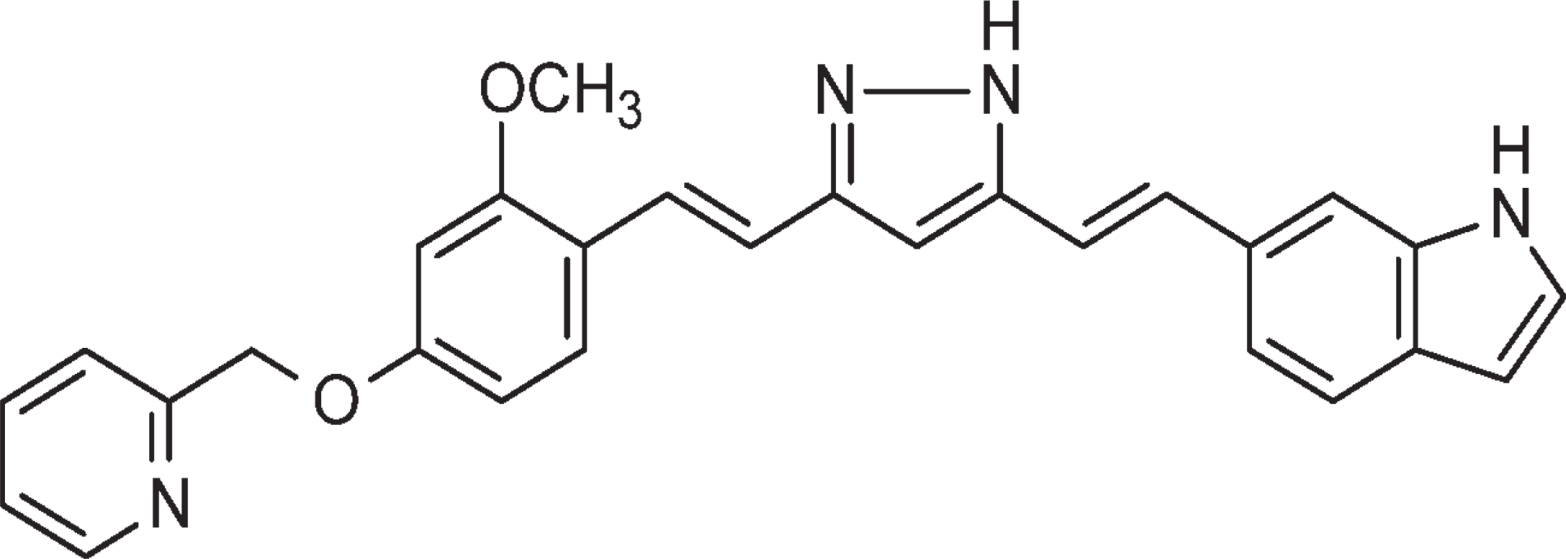
Chemical structure of PE859.

The chemical structure of the test compound 3-[(1E)-2-(1H-indol-6-yl)ethenyl]-5-[(1E)-2-[2-methoxy-4-(2-pyridylmethoxy)phenyl]ethenyl]-1H-pyrazole is shown in Fig. 1. This compound was synthesized in accordance with the procedure described in the patent WO2012141228.

### 2.2. Preparation of recombinant tau protein

*E*.*coli* expressing His-tagged three-repeat microtubule-binding domain (3RMBD) of human tau was kindly provided by Professor T. Ishida [16]. Cell pellets were suspended in 50 mM Tris-HCl buffer (pH 7.6, Wako Pure Chemical, Osaka, Japan) with 50 mM NaCl (Wako Pure Chemical) and sonicated. Supernatants were then purified on an affinity chromatography column. Ni Sepharose 6 Fast Flow (GE Healthcare, Amersham, UK) was filled in a column and 100 mM NiSO_4_ (Nacalai tesque, Kyoto, Japan) was applied on the column. The supernatants were filtered with 0.45 μM Millex syringe-driven filter unit (Merck Millipore, Billerica, MA, USA), applied on the column and eluted with 10 mM, 40 mM, 100 mM and 500 mM of imidazole containing 500 mM NaCl and 50 mM Tris-HCl pH 7.6. The eluted fractions of 100 mM imidazole were dialyzed with 100 mM ammonium acetate at 4°C over night. Protein concentrations of the dialyzed solutions were determined by UV absorption at 280 nm. Full length tau 2N4R human tau was cloned into the pRK172 expression vector, expressed in BL21 (DE3) *E*. *coli*, and prepared according to the previous study [17]. Briefly, *E*. *coli* cells expressing full length tau were homogenized in buffer of 50 mM PIPES, 1 mM EGTA, 1mM DTT containing protease inhibitors. The homogenate was boiled for 15 minutes and centrifuged at 27,000g. Supernatant was purified by ion-exchange chromatography (P11, GE Healthcare), gel filtration chromatography (NAP10, GE healthcare), and reverse phase-high performance liquid chromatography (COSMOSIL 5C8-AR column, Nacalai tesque). After sample was lyophilized, tau was dissolved in water and stocked at -80°C.

### 2.3. ThT fluorescence assay

Tau aggregation was monitored using thioflavin T (ThT) (Sigma Aldrich, St. Louis, MO, USA), a fluorescent dye which binds specifically to beta-sheet structure of protein. The test compound, 10 μM 3RMBD and 10 μM heparin (Sigma Aldrich) were dissolved in 50 mM Tris-HCl (pH7.6), and incubated at 37°C up to 144 hours. At each point of incubation time, 135 μL of the solutions were removed and mixed with 15 μL of 100 μM ThT solution (final concentration: 10 μM) and the fluorescence intensity with excitation at 440 nm and emission at 486 nm was measured. In the assay using full length (2N4R) human tau, 10 μM tau solution with 10mM HEPES (pH 7.4), 100mM NaCl, and 10 μM ThT with or without test compounds was dispensed into 96 well black plate. Tau polymerization was induced by adding 10 μM heparin and incubating at 37°C (final volume: 50 μL per well). These fluorescence intensities were measured with a multilabel counter ARVO (Perkin Elmer, Wellesley, MA, USA) at an excitation wavelength of 444 nm and an emission wavelength of 485 nm. Measurements were carried out at the time points indicated [18].

### 2.4. Transmission Electron Microscopy (TEM) Analysis

Aggregated 3RMBD tau was observed by TEM using an electron microscope JEM-1220 (JEOL, Tokyo, Japan). The tau aggregation reaction mixtures containing 10 μM 3RMBD, 10 μM heparin and PE859 (0, 0.1 or 1 μM) were incubated at 37°C for 24 h, and applied to carbon grids followed by staining with 2% phosphortungstic acid (TAAB laboratories, Aldermaston, UK). The electron microscope was operated at 80 kV and the grid was observed at a ×20,000 magnification.

### 2.5. Circular dichroism (CD) Spectroscopy

The secondary structure of aggregated tau was monitored by circular dichroism (CD) spectroscopic measurement using a J-720 spectrometer (Jasco, Tokyo, Japan). The tau aggregation reaction mixtures containing 25 μM 3RMBD, 25 μM heparin and 10 μM PE859 were incubated at 37°C for 24 hours, followed by the measurement. An optical cell with a path length of 1 mm was used. The spectra from 190 nm to 250 nm at 25°C were measured with a scan speed of 20 nm/min.

### 2.6. Measurement of PE859 concentration in blood and brain after oral administration

Male, 7-week-old ICR mice were obtained from Japan SLC (Hamamatsu, Japan). PE859 was dissolved in 80% PEG 400 (NIKKO Pharmaceutical, Hashima, Japan), 10% HCO40 (Kao, Tokyo, Japan) and 10% water solution at 5 mg/ml, and orally-administered to mice at a dose of 40 mg/kg. The mice were sacrificed under deep anesthesia with pentobarbital (Kyoritsuseiyaku, Tokyo, Japan) at 100 mg/kg, i.p., at 1, 3, 6, 10, 15, or 24 h after administration, and blood was collected from inferior vena cava and brain was isolated. Plasma was separated from blood by centrifugation at 800 g for 10 min at 4°C. Brain samples were homogenized in 3 volumes (weight/volume) of PBS. The level of PE859 in plasma and brain homogenate was measured using LC-MS/MS system ACQUITY UPLC (Waters, Milford, MA, USA) and API 4000 (AB SCIEX, Framingham, MA, USA). Samples were mixed with equal amount of methanol and 3 volume (v/v) of internal standard solution (100 ng/ml warfarin in methanol), and injected into the LC-MS/MS. These analyses were performed at Sekisui Medical Co., Ltd. (Tokyo, Japan). Experimental procedures involving mice and their care were performed in accordance with the Animal Research: Reporting *In Vivo* Experiments (ARRIVE) guidelines for the care and use of laboratory animals. The protocol was approved by the Committee on the Ethics of Animal Experiments of Pharma Eight (Permission Number: 2011–1). All efforts were made to minimize suffering.

### 2.7. PE859 treatment to P301L tau transgenic mice

Male homozygous JNPL3 transgenic mice, expressing human tau with P301L mutation gene, were purchased from Taconic Farms, Inc. (NY. USA). The mice were bred and treated at Institute for Animal Reproduction (Kasumigaura, Japan) during this study. The 98 mice were randomly divided into 2 groups of 49 mice. To conduct the study in a blinded manner, the result of grouping was not disclosed to the people in charge of evaluation of efficacy (western blotting, tail hanging test and rotarod test, described later). PE859 was dissolved in 80% PEG400 and 20% water solution at 5 mg/ml, and orally-administered at a dose of 40 mg/kg/day for 6 months (from 9 to 15 months of age). The body weights of the mice were measured once a week during PE859 treatment. At the end of the study, animals were sacrificed under deep anesthesia with pentobarbital (Kyoritsuseiyaku, Tokyo, Japan) at 100 mg/kg, i.p., perfused by injecting 30 ml of saline from the heart, and spinal cords were removed for biochemical and histochemical analyses. Experimental procedures involving mice and their care were performed in accordance with the Animal Research: Reporting *In Vivo* Experiments (ARRIVE) guidelines for the care and use of laboratory animals. The protocol was approved by the Committee on the Ethics of Animal Experiments of Institute for Animal Reproduction (Permission Number: 24–55). All efforts were made to minimize suffering.

### 2.8. Extraction of sarkosyl-insoluble aggregated tau

In this study, we modified the extraction method of Sahara et al.[19]. Spinal cord tissues were homogenized in 19 volumes (w/v) of extraction buffer containing 50 mM Tris-HCl (pH7.5), 5 mM EDTA (Nippon Gene, Tokyo, Japan), 1 mM EGTA (Nacalai tesque), 1% NP-40 (Sigma Aldrich), 0.25% deoxycholic acid sodium salt (Sigma Aldrich), 0.1 M NaCl, 0.5 mM PMSF (Sigma Aldrich), 1 × PhosSTOP (Roche, Basel, Schweiz), and 1 × Complete EDTA(-) (Roche). Homogenates were centrifuged at 250,000g at 4°C for 20 minutes. The supernatants were collected as the tris buffer-soluble fraction (S1), and 10 volumes (tissue weight/volume) of sarkosyl buffer containing 10 mM Tris-HCl (pH7.5), 0.5M NaCl, 1 mM EGTA, 10% sucrose (Wako Pure Chemical), and 1% sarkosyl were added to precipitates followed by sonication. The solutions were incubated at 37°C for 60 minutes, and centrifuged at 250,000g at 4°C for 20 minutes. The supernatants were collected as the sarkosyl-soluble fraction (S2), and 10 volumes (tissue weight/volume) of PBS (Nacalai tesque) were added to precipitates followed by sonication (sarkosyl-insoluble fraction, P2).

### 2.9. Western blotting (WB) for sarkosyl-insoluble tau

Each fractions were added to 4× NuPAGE sample buffer (Life technologies Carlsbad, CA, USA), heated at 70°C for 10 min, and electrophoresed in 12.5% polyacrylamide gels (Bio Craft, Tokyo, Japan), and then electroblotted to 0.2 μm PVDF membranes (Bio-Rad, Hercules, CA, USA). The blots were blocked with 2.5% skimmed milk (Nacalai tesque) in 1× TBS-T (Sigma Aldrich) for 1 h at room temperature. After blocking, the blots were incubated with a monoclonal anti-tau antibody HT7 (1:1,000, Thermo Fisher Scientific, Waltham, MA, USA) in blocking solution for 2 h at room temperature. The blots were washed with 1× TBS-T for 30 minutes. The washed blots were incubated with HRP-conjugated anti-mouse IgG (1:2000, GE healthcare) for 1 h at room temperature, and washed as described above. Tau proteins were detected by chemiluminescent HRP substrate (Merck Millipore) and analyzed by using an imaging analyzer ChemDoc (Bio-Rad). For preparation of standard curve to calculate the amount of tau, the 2-fold serial dilutions of sarkosyl-insoluble fraction were included in each gel. The standard dilutions were prepared by mixing some of the WB samples, which were preliminarily selected by WB of some severe-clasping phenotype samples. The samples outside the range of the standard curve were properly diluted and re-assayed.

### 2.10. Immunohistochemistry

Tissues were fixed in 4% paraformaldehyde (Wako Pure Chemical) for 24 h and stored in 10% sucrose in PBS at 4°C. Fixed tissues were dehydrated with 70% ethanol, acetone and 100% ethanol, transferred into xylene (10 minutes × 3 times), and paraffin (30 minutes × 3 times, at 60°C), and then embedded in paraffin. Paraffin-embedded tissue blocks were cut into 10 μm thick sections using a sliding microtome (Nippon Optical Works, Tokyo, Japan), and extended on MAS-coated glass slides (Matsunami Glass, Kishiwada, Japan). After drying at 60°C for 24 h, the sections were deparaffinized with xylene (10 minutes × 3 times) and ethanol (100%, 90%, 80% and 70%), washed with PBS, and then stained with antibodies against phosphorylated tau at Ser/Thr 202/205 (AT8, 1:100 dilution; Thermo Fisher Scientific) or neuron (NeuN, 1:100 dilution; Millipore), using M. O. M Immunodetection Peroxidase kit (Vector Laboratories, Burlingame, CA, USA). 3,3’-diaminobenzidine (DAB, Wako Pure Chemical) at a concentration of 0.5 mg/ml in PBS with 0.005% hydrogen peroxide was used as chromogen. The sections were dehydrated with ethanol (70%, 80%, 90%, and 100%) and xylene, and then mounted with mounting medium (Daido Sangyo, Tokyo, Japan). The sections were observed using a microscope BZ-8100 (Keyence, Osaka, Japan)

### 2.11. Tail hanging test

The mice were monitored for the condition of the hind limbs weekly. To evaluate the condition, mice were lifted at the tail and hung at the height of 10 cm from the cage. Normally, the healthy mice can open their hind limbs to both sides. But JNPL3 mice with advanced tau pathology cannot open hind limbs and cannot move enough, and these conditions were defined as the onset of motor dysfunction.

### 2.12. Rotarod test

The mice were placed on a rotating rod of a Rotarod treadmill MK-670 (Muromachi kikai, Tokyo, Japan), which was slowly accelerated to 40 rounds per minute in 180 seconds. The latency to fall from the rotarod was recorded for a maximum of 180 seconds. Mice were given two trials each day for 3 consecutive days at the first 3weeks and 2th, 4th and 6th months of administration, respectively. The trial of first and second day was regarded as training, and the average time of two trials for the third day was used for analysis.

### 2.13. Data analysis

The comparison of WB data between the vehicle group and the PE859 treatment group was performed using Mann-Whitney tests. Mortality and the onset of motor dysfunction in the tail hanging test of each group were indicated in Kaplan-Meier curve and analyzed using log-rank test. The fall latency of rotarod test and the body weight of the two groups were compared using two-way repeated measures analysis of variance (RM ANOVA) and post-hoc Bonferroni test. The software Graphpad Prism was used to perform these statistical analyses, and p < 0.05 was considered significant.

## Results

### 3.1. PE859 inhibits tau aggregation in a concentration-dependent manner

**Fig 2:**
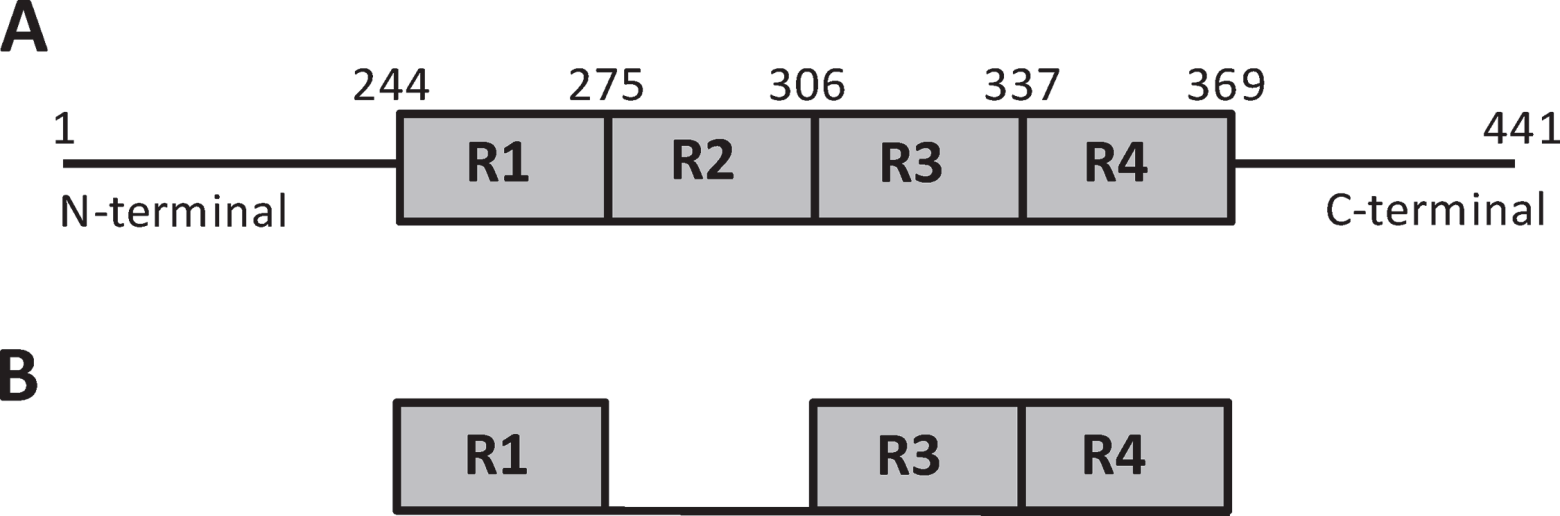
Tau constructs used in *in vitro* assay. (A) Full length tau. It is the longest tau isoform with 441 amino acids, which includes four microtubule binding domains. (B) 3RMBD tau peptide, lacking N-terminal region, the second microtubule-binding domain (R2), and C-terminal region.

To examine the inhibitory effect of PE859 against tau aggregation *in vitro*, we used two kinds of constructs of tau protein (Fig. 2). Full length human tau consists of 441 amino acids, which has four microtubule-binding repeat domains (Fig. 2A). 3MBD peptide (Fig. 2B) has only 3 repeat domain.

**Fig 3:**
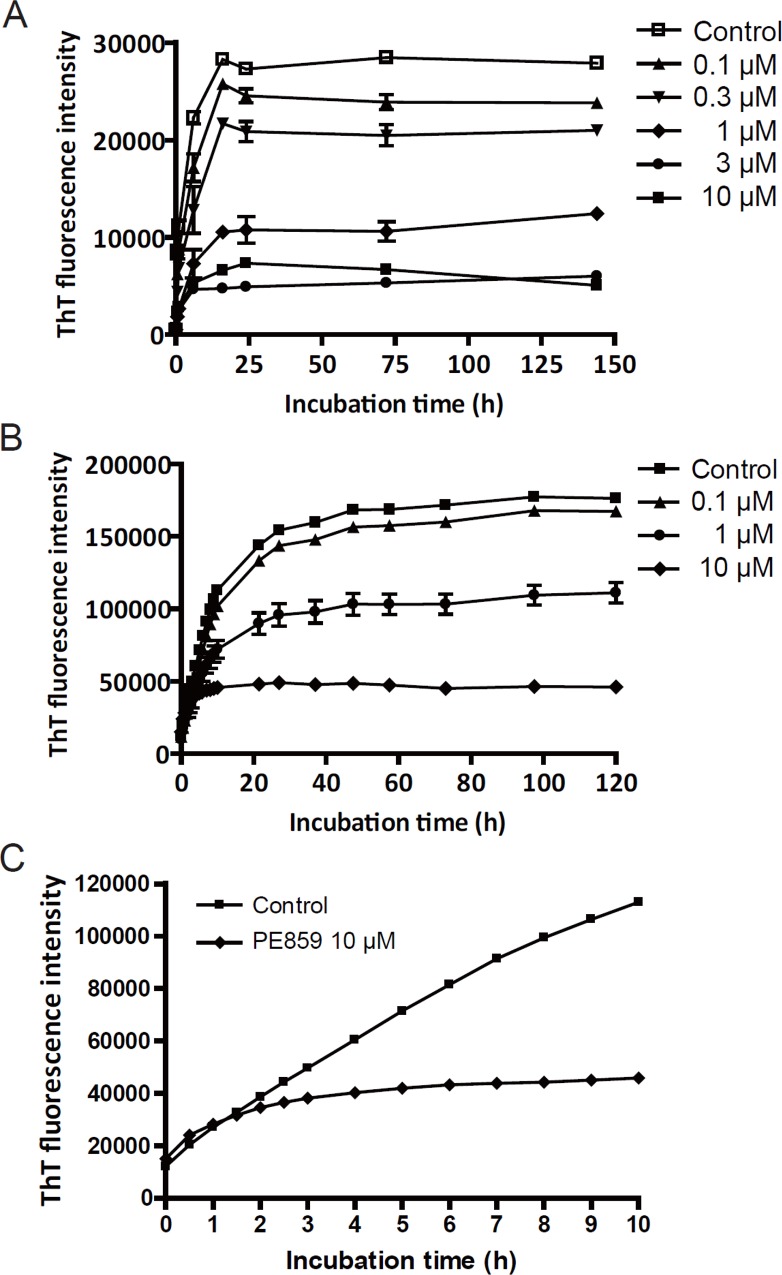
Inhibitory effect of tau aggregation induced by PE859. (A) The time course of aggregation of 3RMBD Mean ± SEM, n = 5. (B) The time course of aggregation of full length tau. (C) The enlarged display of time course of aggregation of full length tau monitored by ThT fluorescence, that is the selected data of control and PE859 10μM up to 10 h from Fig. 3B. Mean ± SEM, n = 3. The aggregation of these proteins was monitored by ThT fluorescence.

The aggregation of the repeat peptides of tau can be monitored by the fluorescence intensity of the thioflavin dyes [20]. Thus, we used ThT to monitor the profile of inhibition of tau aggregation of PE859 (Fig. 3). Firstly, we carried out the primary screening using 3RMBD in order to select the compounds capable to inhibit tau aggregation. And then the inhibitory capacity of selected compounds against tau aggregation was confirmed using full length tau. PE859 inhibited the heparin-induced aggregation of both 3RMBD and full length tau in a concentration-dependent manner. In each assay, the IC_50_ values calculated at the last measurement periods were 0.81 μM, and 2.23 μM, respectively. At the beginning of the incubation, there was little difference in ThT fluorescence between control and PE859 (Fig. 3C). But, after 1 h incubation, the increase in ThT fluorescence of the sample with PE859 was drastically inhibited and reached a plateau at 10 h.

**Fig 4:**
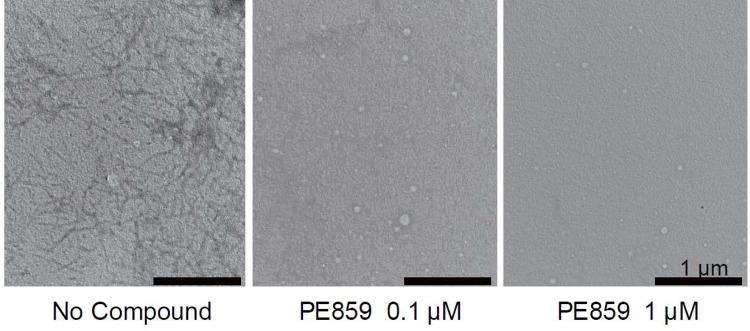
Inhibiton of tau aggregation by PE859 was observed using by transmission electron microscopy. 10 μM 3RMBD with no compound (left) and with 0.1 μM PE859 (center) or 1 μM PE859 (right) were incubated at 37°C for 24 h, and stained by 2% phosphotungstic acid. Scale bar = 1 μm.

The results of the ThT fluorescence assay were confirmed with other methods. In transmission electron microscopy (Fig. 4), 3RMBD tau with no compound formed fibrous aggregates after 24 h incubation. However, tau aggregates were scarcely observed in the sample with PE859.

**Fig 5:**
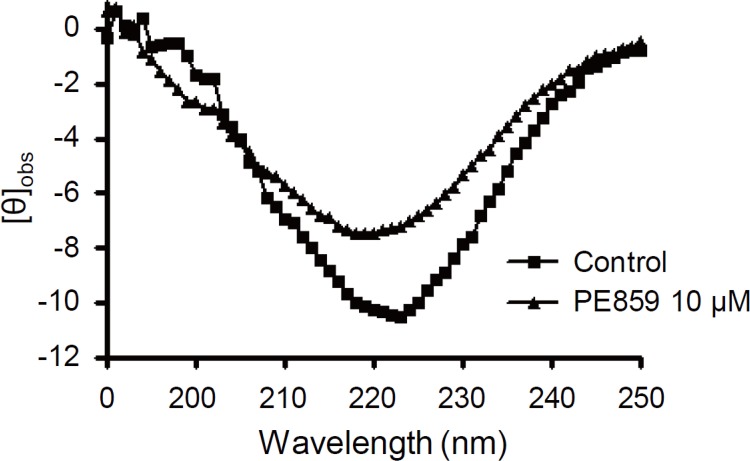
The CD spectra of the aggregated tau. 25 μM 3RMBD in the absence of PE859 (square) and presence of 10 μM PE859 (triangle) were incubated at 37°C for 24 h.

In CD spectroscopic measurements (Fig. 5), the peak at about 220 nm wavelengths, which indicates the formation of beta-sheet structure, was detected in the absence of PE859. But in the presence of PE859, the peak became smaller. These results suggest that PE859 inhibits tau aggregation through formation of beta-sheet structure.

### 3.2. Orally administered PE859 is distributed to blood and brain in mice

**Fig 6:**
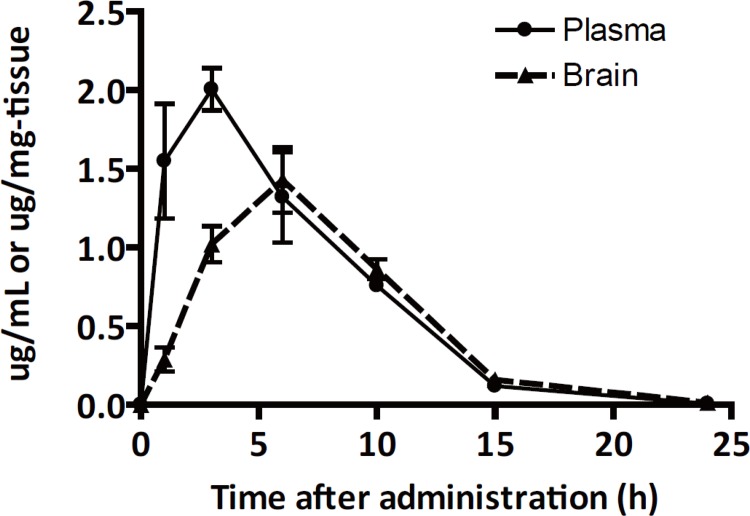
The concentration of PE859 in plasma and brain after an oral administration. The concentration of PE859 in plasma and brain after an oral administration to male ICR mice at 40 mg/kg, were showed by circles with straight line and triangles with dot line, respectively. Mean ± SEM, n = 4.

We determined whether orally administrated PE859 reaches the central nervous system (CNS) tissues. PE859 was orally administrated to mice at 40 mg/kg and the time-dependent change of PE859 concentration in the blood and the brain were measured (Fig. 6). The maximum concentration of PE859 was 2.005 ± 0.267 μg/ml in the blood at 3 h and 1.428 ± 0.413 μg/g in the brain at 6 h. At 24 h, PE859 was slightly detected in both regions (0.008 ± 0.004 μg/ml in blood and 0.014 ± 0.004 μg/g in brain). The AUC calculated from each graph was 16.24 μg·hr/ml and 13.03 μg·hr/ml respectively, and the brain-plasma ratio was 0.80. These results suggest that PE859 could cross the blood-brain barrier and that PE859 could be distributed into the tissues of the central nervous system.

### 3.3. PE859 delays onset and progression of the motor dysfunction in JNPL3 mice

**Fig 7:**
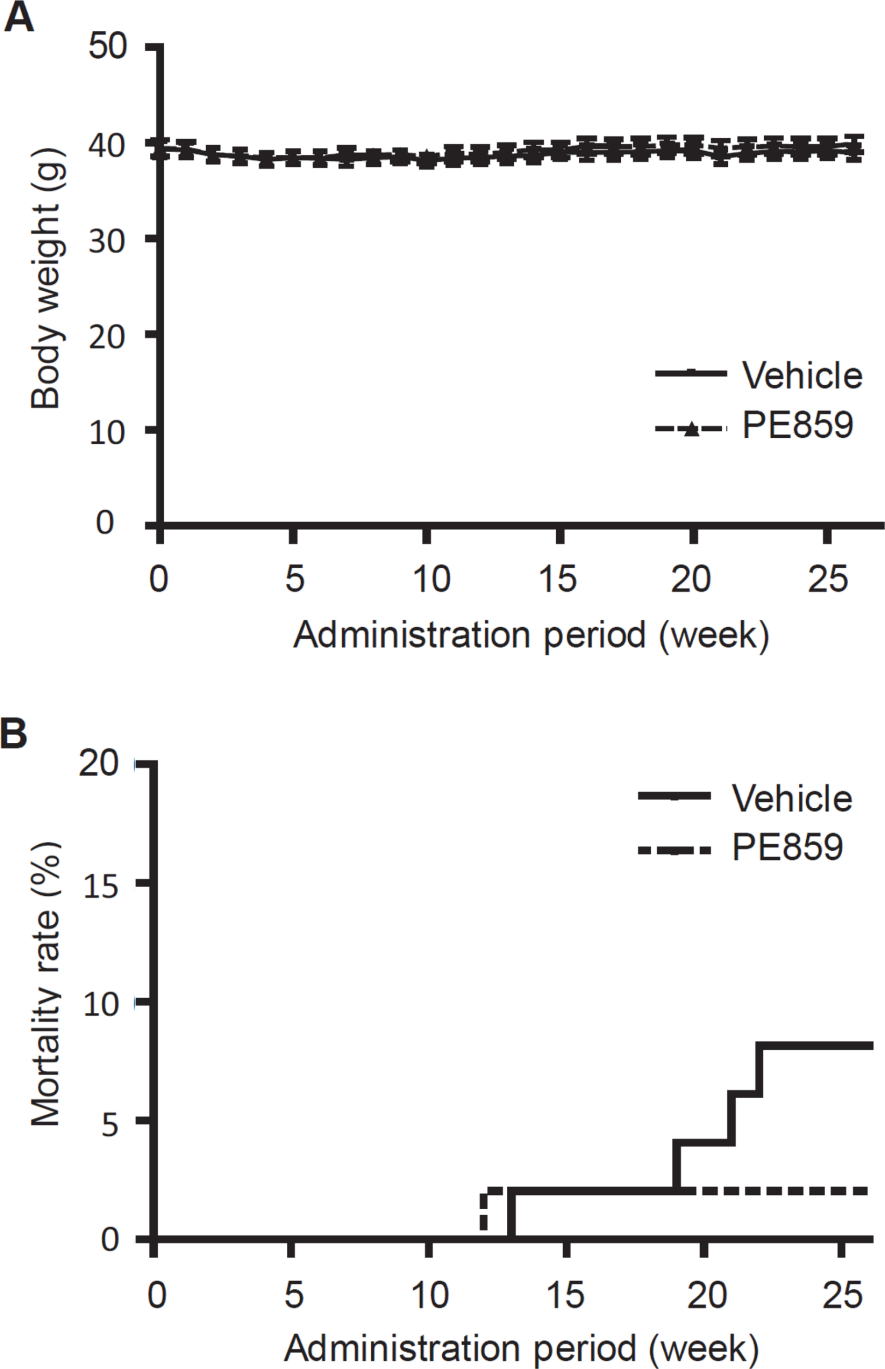
The body weight and mortality rate. (A) The average body weights of vehicle (squares with straight line) and PE859 treatment group (triangles with dot line) was indicated by mean ± SEM. There was no significant difference between treatment groups (p = 0.71; two-way RM ANOVA). (B) The mortality rate of vehicle (straight line) and PE859 treatment group (dot line) were indicated by survival curve. The rates of dead mice during this study were 8.2% (4/49) for the vehicle group and 2.0% (1/49) for the PE859 treatment group, and there was also no significant difference between the two groups (p = 0.18; log-rank test).

In this study, we treated PE859 to JNPL3 mice at 40 mg/kg/day for 6 month. During the treatment, there were no significant change in the body weight between vehicle group and PE859 treatment group (p = 0.71; two-way RM ANOVA, Fig. 7A), and there was no abnormal findings unique to the PE859 treatment group both in appearance and in internal organs (data not shown). These results suggest that PE859 is well tolerated in the conditions of this study. During the treatment period, four mice died in the vehicle group and one mouse in the PE859 treatment group. All the dead mice showed severe motor dysfunction which was characteristic of JNPL3 mice. There was no significant difference between the two groups in the mortality rates (p = 0.18; log-rank test, Fig. 7B).

**Fig 8:**
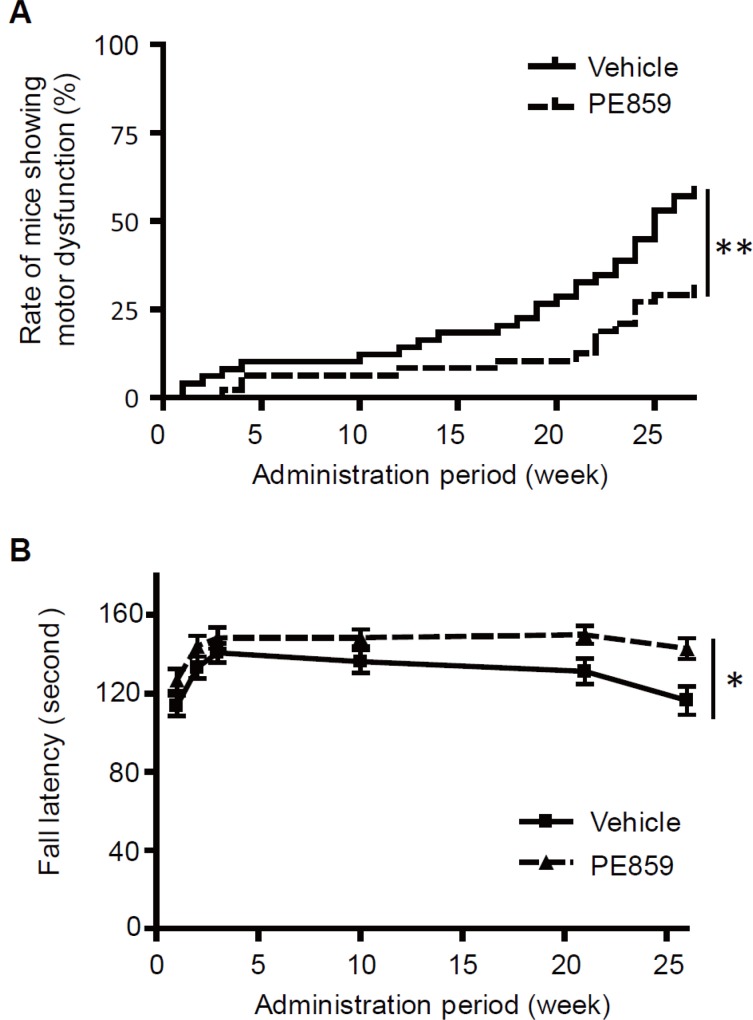
Tail hanging test and rotarod test. (A) The number of mice showing motor dysfunction in the tail hanging test was shown by survival curve. The rates of motor-dysfunctional mice at the end of the study were 57.1% (28/49) for the vehicle group and 30.6% (15/49) for the PE859 treatment group. The difference between the two groups was significant (p = 0.006; log-rank test). (B) The fall latency of rotarod test. Mean ± SEM, n = 45∼48. The data of dead mice were eliminated because of statistics analysis. The difference between the two groups was significant (p = 0.013; two-way RM ANOVA). *p<0.05, and **p<0.01.

JNPL3 mice show motor dysfunction in advance of learning and memory impairment due to the tau accumulation in spinal cord. Progression of tauopathy should be estimated by the degree of motor dysfunction. To monitor the motor dysfunction of the mice, we checked the condition of hind limbs of the mice during administration (Fig. 8A). At the start of administration, all the mice could normally open their hind limbs. And at the end of administration, the rates of the dysfunctional mice, that could not open and move their hind limbs, were 57.1% (28/49) for the vehicle group and 30.6% (15/49) for the PE859 group, resulting in the significant difference (p = 0.006; log-rank test). We also performed rotarod test (Fig. 8B). The mice were trained for the first 3 consecutive weeks of administration, and then checked at the second, fifth, and sixth months. At the third week of administration, the fall latency was 138.7 ± 4.8 seconds for the vehicle group and 147.4 ± 5.3 seconds for the PE859 group. In the vehicle group, the rotarod performance declined consistently, but, in the PE859 treatment group, the rotarod performance did not decline so much, showing significant difference between the two groups (p = 0.013; two-way RM ANOVA). At the end of administration, the fall latency was 116.4 ± 7.2 seconds for the vehicle group and 142.8 ± 5.4 seconds for the PE859 group, resulting in significant difference (p<0.01; Bonferroni posttests). These results suggest that PE859 delays onset and progression of the motor dysfunction in JNPL3 mice.

### 3.4. PE859 reduces the amount of sarcosyl-insoluble tau in the spinal cord of JNPL3 mice

**Fig 9:**
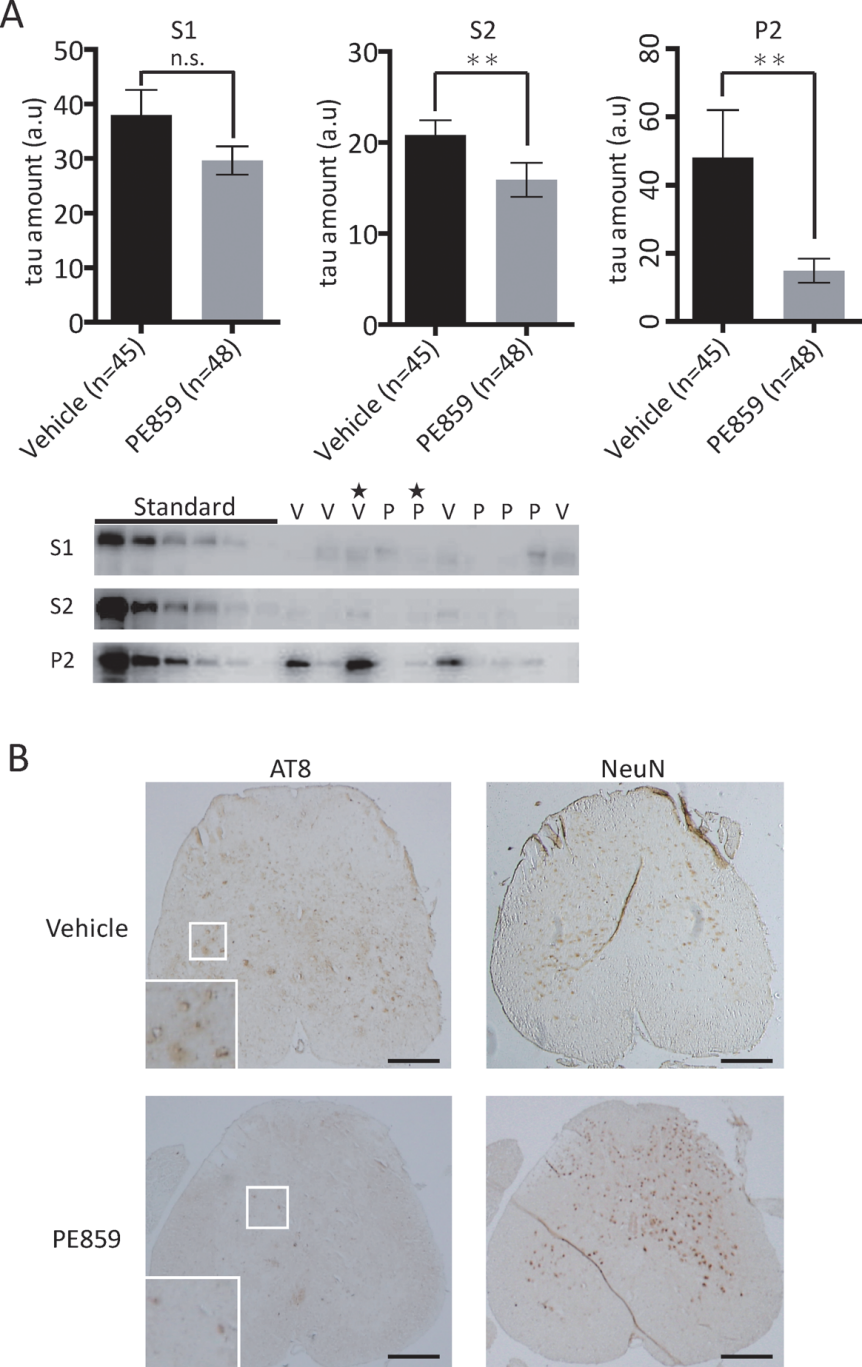
Western blotting and immunohistochemistry. (A) The amounts of tris buffer-soluble tau (S1), sarkosyl-soluble tau (S2) and sarkosyl-insoluble tau (P2) in the spinal cord. The amount of sarkosyl-soluble tau and sarkosyl-insoluble tau of the PE859 group were significantly smaller than those of the vehicle group (p = 0.009 and 0.008, respectively; Mann-Whitney test). The data were calculated using by the standard curve, which was prepared by the 2-fold serial dilutions of the P2 samples as the pictures of WB (lanes of “Standard”). The lane “V” or “P” of the WB picture means Vehicle or PE859 treated mouse, respectively. Mean ± SEM. a.u., arbitrary unit. **p<0.01. (B) The representative photomicrographs of immunostaining against aggregated tau (AT8) and neuron (NeuN). The spinal cords of the two mice starred in the WB picture of Fig. 9A were used. Scale bar, 0.2 mm.

The amount of tris buffer-soluble tau (S1 fraction), tris buffer-insoluble but sarkosyl-soluble tau (S2 fraction) and sarkosyl-insoluble tau (P2 fraction) in spinal cord is shown in Fig. 9A. The mean ± SE of the relative amounts of sarkosyl-insoluble tau was 48.0 ± 13.9 for the vehicle group and 14.9 ± 3.5 for the PE859 group, resulting in significant difference (p = 0.008; Mann-Whitney test). The relative amounts of tris-soluble tau was 37.9 ± 4.6 for the vehicle group and 29.6 ± 3.0 for the PE859 group, there was no significant difference (p = 0.29). The relative amounts of sarkosyl-soluble tau was 20.8 ± 1.6 for the vehicle group and 15.9 ± 1.8 for the PE859 group, resulting in significant difference (p = 0.005). PE859 delayed progression of the motor dysfunction through the inhibition of accumulation of sarkosyl-insoluble tau. Representative photomicrographs of immunostaining with antibodies against phosphorylated tau (AT8) or neuron (NeuN) in the spinal cord are shown in Fig. 9B. In PE859 treated mouse with low level of insoluble tau in WB, the intensity of AT8 signal was lower than that of vehicle mice with high level of insoluble tau. And the number of NeuN-positive cell in PE859 treated mouse was larger than that of vehicle mouse.

## Discussion

The compound PE859, which was selected from our original library compounds, inhibited the aggregation of both 3RMBD and full length (2N4R) tau in concentration-dependent manner *in vitro* assays (Fig. 3). The IC_50_ value for 3RMBD was about one-third of that for full length tau (0.81 μM and 2.23 μM, respectively). Full length tau contains four-repeat microtubule-binding domain (4RMBD) in its sequence, and the aggregation profile and conformational change of 4RMBD is thought to differ from those of 3RMBD [21]. According to the study, 4RMBD has two cysteine residues and it can form an intramolecular disulfide bond. On the other hand, 3RMBD has only one cysteine residue, so it forms intermolecular disulfide bond. This difference in amino acid sequence and early formation between the two kinds of tau proteins might contribute to the difference in the IC_50_ values. C-terminal and N-terminal sequences of full length tau might also decrease the accessibility of PE859 to MBD and hinder the tau aggregation inhibition of PE859.

In human CNS tissue, the condition of tau aggregation is not so simple like *in vitro* assays. In human brain, six different isoforms of tau are expressed *via* alternative splicing of the MAPT gene. In addition, tau proteins are subject to proteolytic cleavage, and these tau fragments are detected in tauopathy brain [22–24]. They may form the PHF core that promotes aggregation of tau including other tau species [25–27]. Various post-translational modifications other than phosphorylation, such as nitration, glycosylation, acetylation, glycation, ubiquitylation [28], and O-Glc NAcylation [29], also have an effect on tau aggregation. It is also reported that RNA can binds to tau and enhances tau aggregation [30]. Therefore, it is very difficult to replicate the condition of aggregation actually occurred in human brain *in vitro* assay, and the research confirming the efficacy *in vivo* is also required.

An orally administrated PE859 was absorbed into the blood and 80% amount of PE859 in blood transferred into the brain (Fig. 6). This result suggests that PE859 has blood-brain barrier permeability, which is desired for the drug of CNS diseases.

For *in vivo* study, we selected JNPL3 mice. These mice express the human tau with P301L mutation, which is associated with FTDP-17. Comparison of insoluble tau from JNPL3 mice with that from human FTDP-17 brains shows a similarity in the WB band pattern [31]. PHF of FTDP-17, AD and other tauopathies are reported to consist of similar set of tau species [32]. These findings suggest that similar tau pathology occurs in both human tauopathies and in JNPL3 mice.

As a result of *in vivo* study, the amounts of sarkosyl-insoluble tau and sarkosyl-soluble (but tris buffer-insoluble) tau in PE859 treated mice significantly decreased, but the amount of tris-soluble tau was not different between two groups (Fig. 9A). These results suggest that PE859 showed inhibitory effect on tau aggregation in the spinal cord. In addition to the effect on the tau pathology, PE859 also improved the motor dysfunction (Fig. 8). These results suggest that the inhibitory effect of PE859 on tau aggregation in the spinal cord resulted in the improvement of the motor function. Furthermore, neither weight loss nor abnormal findings attributable to drug toxicity were observed in PE859 treatment group during the 6-month administration (Fig. 7A). It demonstrates the safety and tolerability of oral chronic administration of PE859.

Methylene blue (MB) is a well-known tau aggregation inhibitor [13, 33]. Recently, the mechanistic basis of MB on tau aggregation inhibition was reported [34]. Phase 2 clinical trials for MB in AD patients had been completed and positive results were obtained [35]. LMTX, a reduced form of MB was announced to be tested in the phase 3 clinical trials. On the other hand, recent *in vivo* study using JNPL3 mice reports that MB induced autophagy and reduced total tau level, not insoluble tau level [36]. Another study on JNPL3 mice also shows that MB reduced hyperphosphorylated tau in insoluble fraction. However, the total amount of insoluble tau did not differ [37]. In the study using other tau transgenic mice rTg4510, MB did not affect pathology with tangle formation and neurodegeneration [38]. MB might not have been proven to be efficacious as a tau aggregation inhibtor at least in *in vivo* study.

Meanwhile, recent studies suggest that the oligomeric aggregates of tau may be associated with tau-mediated neurotoxicity [37–40]. In clinical data, cognitive decline correlated with pretangle pathological events before deposition of frank NFTs [41]. A computer program suggested that hippocampal neurons having NFT lived for up 20 years in AD [42]. In rTg4510 mice expressing human P301L tau in various forebrain areas under Tet-off system, progressive age-related NFT formation, neuronal loss and behavioral impairment was observed. Suppression of tau transgene by doxycycline treatment in the rTg4510 mice prevented neuronal loss and behavioral impairment although NFT continued to grow [43]. Overexpression of FKBP51 by AAV9 injection increased tau oligomer levels inducing neuronal toxicity in transgenic mice [44]. The injection of tau oligomer, not monomer or fibril, into normal mouse brain causes memory impairment with synaptic and mitochondrial dysfunction [45]. In the mice expressing human wild-tau with deletion of endogenous tau, injection of anti-tau oligomer antibody decreased tau oligomer levels and improved cognitive impairment [46]. The precise mechanism of tau-mediated neurotoxicity remains to be clarified. In our ThT fluorescence assay, PE859 mainly inhibited the tau aggregation reaction occurred in 1–10 h incubation period (Fig. 3C). According to the previous study on the same 2N4R type of tau [17], ThT fluorescence level increases at 4h incubation, along with the formation of granular-shaped 40mer tau. And then, during 6–21 h incubation period, the granules rapidly increase and they further form PHF gradually. PE859 might inhibit tau aggregation at the stage of oligomer or granule formation.

We expect that PE859 inhibits tau aggregation at the stage prior to the generation of its neurotoxic form, and consequently sarkosyl-insoluble tau in JNPL3 mice also decreases. Further study on the detail molecular mechanism for tau aggregation inhibition of PE859 will lead to the elucidation of the mechanism of tau-mediated neurotoxicity.
